# The Questions, Challenges, and Possibilities When Joining Critical Disabilities Studies and Healthcare Research

**DOI:** 10.3390/healthcare13222925

**Published:** 2025-11-15

**Authors:** Madelyn Toman, Meredith Atanasio, Pamela B. Teaster

**Affiliations:** Department of Human Development and Family Science, Virginia Polytechnic Institute and State University, Blacksburg, VA 24060, USA; matanasio@vt.edu (M.A.); pteaster@vt.edu (P.B.T.)

**Keywords:** critical disability studies, healthcare and medical research, interdisciplinary research

## Abstract

**Background/Objectives:** Interdisciplinary research teams that include critical disability studies (CDS) scholars and Healthcare and Medical Researchers have the potential to investigate complex lived experiences and explore new opportunities to best serve disabled communities. However, individuals in these fields typically approach disability research in different ways. Throughout this manuscript, we refer to a hypothetical interdisciplinary research team as an example of how to integrate the questions, challenges, and possibilities into practice when joining CDS and Healthcare and Medical Research. **Discussion:** First, we raise three large and complex questions that researchers must address (and discuss) when conducting disability research: (a) what is (a) disability, (b) what does it mean to live with a disability, and (c) who is included in research samples/as research participants for disability research? Then, we discuss the colliding and harmful relationship history between CDS and Healthcare and Medical Research fields, and the continued oppositional training of professionals in both fields. Finally, we offer insights into how collaborative efforts and methods of interdisciplinary research teams can optimize success when tackling complex research questions to serve disabled communities. **Conclusions:** We suggest approaches for projects at the intersection of CDS and Healthcare and Medical Research: holistic, person-centered research, treating individuals in the disability community as experts, and collaborating with the community while conducting research. This manuscript serves as a starting point for researcher teams looking to conduct ethical, rigorous, and trustworthy research at the intersection of health, medicine, and disability.

## 1. Introduction

Interdisciplinary research is a popular approach to social science research [1,2]. When members of research teams have different disciplinary backgrounds and approaches, they can tackle complex, unexplored, or underexplored questions. Although interdisciplinary work can be exciting and is often desirable for academics, providers, funders, and policy makers, it is not necessarily easy or successful.

Challenges can arise when researchers attempt to bring together fields of research that do not naturally mix well. One example is the research fields of critical disability studies (CDS) and Healthcare and Medical Research—even though researchers in these fields serve the same population and ask similar questions, their approaches to research and knowledge come from different points of view (and are even at odds).

Throughout this article, we ask readers to reflect on the following research team scenario:

*A team of four researchers have come together to investigate the symptoms, characteristics, and support needed for individuals with Down Syndrome who are diagnosed with early onset Alzheimer’s disease (EOAD). Two of the team members are trained in CDS, one member is an MD/PhD specializing in research on neurodegenerative disorders, and the fourth team member is a physician.*

Below we discuss the types of questions and challenges a research team such as this must address before designing a study as well as possibilities and opportunities that can be achieved when bringing together individuals with multiple points of view and expertise. The purpose of this manuscript is to offer suggestions for beginning a conversation between researchers, to provide examples of where clashes and tensions lay between CDS and Healthcare and Medical Research and offer concrete collaborative solutions for research teams.

## 2. Brief Overview of CDS and Healthcare and Medical Research

Combining the fields of CDS and Healthcare and Medical Research brings together contrasting “camps” or research fields, including laboratory, clinical, and epidemiological research and interventions. Healthcare and Medical Researchers often utilize physiological, behavioral, and participant self-report assessments, and engage in longitudinal methods to measure change in health status or disease trajectory over time [3]. The ultimate goal of medical research is to understand the general experience and trajectory of the patient and subsequently develop, test, and determine courses of care (e.g., pharmaceutical, physiological, psychological, etc.) to improve short- and long-term health outcomes [4]. In short, medical research has historically involved identifying clinical signs within a group of people and developing solutions to improve quality of life. However, newer approaches in medical research may make collaboration with CDS researchers more feasible, such as an emerging emphasis on patient health literacy that promotes patient agency and incorporates them into decision-making processes in both medical practice and clinical research [5].

The field of CDS is guided by Critical Disabilities Theory, as well as other critical perspectives such as feminist, crip, and queer theories [6,7,8]. CDS moves beyond relying on models that place disability within or outside of the individual, instead using a human rights approach that argues for equal access to all aspects of social life [9,10]. The “critical” in CDS translates to challenging structures, policies, and social norms that have worked in the past and that continue to oppress present and future generations of individuals with disabilities.

## 3. Questions

Based on the research team scenario described above, consider the primary conversations between the four scholars who are well-versed and trained in their respective fields. Although they may have similar motivations, research questions of interest, and overlapping general interests, the team members are likely to have vastly different approaches to research and service for disabled communities. In this section, we explore how these two “camps” of research respond to seemingly simple questions, yet in ways that are incongruent.

### 3.1. What Is (A) Disability?

As emphasized by influential disability rights activist Judy Heumann, disability is a normal part of life and a key aspect of the human experience [11]. Organizations in the United States, such as the Centers for Disease Control, typically base their broad definitions of disability on that of the World Health Organization, which states:

*Disability is part of being human. An estimated 1.3 billion people—about 16% of the global population—currently experience significant disability. This number is increasing in part due to population ageing and an increase in the prevalence of noncommunicable diseases. Disability results from the interaction between individuals with a health condition, such as cerebral palsy, Down syndrome and depression, with personal and environmental factors including negative attitudes, inaccessible transportation and public buildings, and limited social support.*

[12]

Definitions of disability, like the one above, are typically broad and are highly subjective, reasons why individuals writing, teaching, and researching the topic of disability often rely on models of disability. Depending on the model used, the term disability can hold different meanings. Models of disability offer different ways to conceptualize disabilities, each model with its own strengths and weaknesses. While recognizing that CDS scholars intentionally look beyond only using models of disability to conceptualize and advocate for disability rights and research, the models offer a starting point for a conversation about the meaning of disability.

#### Models of Disability

**Figure 1:**
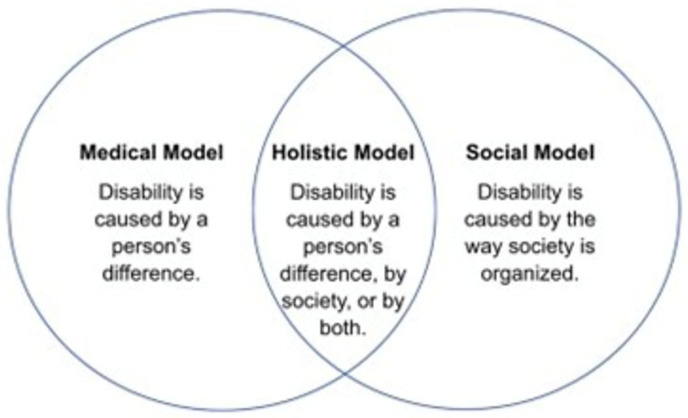
Model of Disability.

The medical model of disability has the longest history and has two key components: pathology must be present, and pathologies are located within individuals [13] (Figure 1). This model reflects an understanding of disabilities as illnesses or diseases that individuals must overcome. With the goal to eradicate symptoms or traits of a disability, the medical model seeks to cure and ‘normalize’ individuals with disabilities [14]. This model has been useful in diagnosing and providing practical care for different types of disabilities, although there are clear weaknesses and shortcomings. For instance, pathologizing disability stigmatizes disabled communities and prioritizes the disability itself (or, more specifically, the diagnosis) over the individual. Furthermore, not all people with disabilities require care or desire cures.

In 1970s, disabled communities began questioning and rejecting the medical model, thus, the social model of disability was developed as an alternative way to conceptualize disability [15]. The social model identifies a marked difference between impairment and disability. Individuals have/are diagnosed with impairments, and disability occurs when social practices and structural environments create barriers and disadvantage individuals with impairments [16,17]. Consequently, society plays a significant role in creating disability through stratification, determining hierarchies, and constructing societal norms [18]. While the social model of disabilities has been a useful tool for social-political movements and gaining civil rights for disabled populations, challenges or weaknesses to this conceptualization of disability exist [17,19]. For example, the social model of disabilities minimizes the embodiment of disability and overestimates the ability of societies to remove all barriers to individuals.

The medical model and the social model of disabilities have been the most prominent approaches that researchers, practitioners, activist groups, and politicians have used to conceptualize disability [20]. There are, however, alternative models of disability, such as the holistic model, economic model, charity model, and the social identity/cultural affiliation model [20,21]. Each offers different definitions of disability and informs policy, perceptions of needs, and disability identity. For the purposes of this manuscript, we describe the two most pertinent to the disability research field while acknowledging and highlighting the complexity that comes with conceptualizing what (a) disability is.

Conceptualizations of disability can use one particular model or a combination of models based on the researchers’ interpretations and personal alignments. Although it is not necessary that all research team members (and researchers broadly) have the same conceptualization of disability, it is the responsibility of individuals conducting research at the intersection of CDS and Healthcare and Medical fields to be transparent and thorough in their descriptions of what disability means to them and in the context of their work.

It is also important to note that CDS scholars go beyond the models of disability when conceptualizing disability. In CDS research, disability is analyzed as a “cultural, historical, relative, social, and political phenomenon” [8]. Research centering disability in CDS highlights narratives of disabled communities and interprets lived experiences of disabled people to deconstruct and challenge binary assumptions of normality vs. abnormality. In doing so, CDS approaches disability through a social justice, person-centered framework.

### 3.2. What Does It Mean to Live with a Disability?

Once individuals identify with and/or are diagnosed with a disability, different types of support and services can be accessed. Not all disabilities are the same—there are many marked differences between an individual’s disabilities (e.g., characteristics, symptomatology, presentation, outcomes). People with the same disabilities may have completely contrasting embodiments and experiences. Medical perceptions of disabilities do not necessarily align with ways that disabled communities view them [22].

An understanding of what it can mean to live with a disability is only possible when people with disabilities are sharing their understandings, stories, and perspectives with researchers, medical providers, policymakers, and others with the power to present disabled narratives. Thus, to truly understand what it means to live with a disability, critical qualitative methodologies, like narrative analysis, community participatory analysis, and phenomenology, should be used by research teams as opportunities to shift from clinical understanding of disability to experiential, contextual understandings of disability.

Individualization in lived experience with disabilities challenges medical discourse around “expectations” and “limitations” frequently explained when receiving diagnoses. Too often, medical professionals provide diagnoses and life expectations as warnings or limited expectations compared to others without those diagnoses. Examples of these types of conversations can be found in the narratives of adults with lifelong disabilities and families [23,24]. This is not to say that medical professionals should not offer knowledge and information about characteristics of diagnoses and a life with specific types of disabilities; however, all information provided to individuals (and families) about what living with a disability could mean upon diagnosis should be based on experiences of people who have similar diagnoses rather than perceptions made by able-bodied people.

### 3.3. Who Is Included in Research Samples/as Research Participants for Disability Research?

Who is included as research participants should depend on the research question(s) and outcome(s) being sought. These considerations have not always been the case. Disability research has been historically dominated by non-disabled voices, including those of medical providers, caretakers, and family members [25]. Research fields risk misrepresentation when they do not include the voices and experiences of the population they serve.

To combat the issue of misrepresentation in research, the popular disability social rights mantra, “Nothing About Us Without Us,” should be extended to researchers intending to serve disabled communities. Disabled communities have expressed the need for inclusive research on disabilities where people with disabilities are more than just participants—they should also serve as consultants, experts, and partners during the research study [25]. Including disabled participants in disability health research allows medical providers and researchers the opportunity to work in partnership with disabled communities and enables the capability to meet their actual needs as opposed to their perceived needs by others [26]. Only by including participants with disabilities in disability research will it be possible to better understand the complexities, barriers, and lives of people with disabilities.

## 4. Challenges

After considering a few fundamental questions and working to consider both points of view, it is also necessary to understand the historical context and current challenges that face researchers aspiring to contribute meaningfully to intersectional work between CDS and healthcare. The history between the medical field and disabled communities has deep-rooted ableist scars and themes of dismissal and misrepresentation that go well beyond the brief description below [27,28]. Nonetheless, we offer specific instances of historical collision and difference in training between medical professionals and disabled communities in order to advance a conversation or explanation of why it is both difficult yet possible for these groups to meet in the middle, or compromise, when coming together in research arenas.

### 4.1. Relationship History

Historically, diagnoses and disabilities have been used against individuals with disabilities in the United States to justify discriminating, segregating, rejecting, and oppressing entire disabled populations [29]. Policies, statutes, and laws in the United States have conspired to strip people with disabilities of their civil and human rights. For example, the verdict from Buck vs. Bell [30] permitted involuntary sterilization of individuals deemed to be “feebleminded” and Ugly Laws in cities across the United States prohibited people with disabilities from appearing in public spaces [31].

Practices put forth by medical professionals (and political desires), such as eugenics and forced sterilization, have dehumanized disabled populations globally. Eugenic ideals and policies, practiced at a national and international level, have targeted disabled communities directly due to the general goal to reduce and eliminate “undesirable” traits for future generations [32,33]. Purposefully and unwittingly, medical and health professionals contributed to the discriminatory, ableist eugenics movement by “furnishing notions of health, diagnosing individuals with defects, and recommending and performing the sexual sterilizations” [33] (p. 3). Newer scientific advancements that serve medical fields, such as genetic testing [34], continue to promote similar ableist thinking. For instance, prenatal screening and genetic testing can be helpful when trying to know and understand more about the fetus; however, they become problematic and ableist in nature when used to identify and eliminate fetuses with disabilities.

The messy and frequently harmful relationship between medical fields and disabled communities is a significant contributor to the deep mistrust between individuals with disabilities and medical researchers [35]. Therefore, it is necessary to increase trustworthiness between researchers and the disabled community in order to conduct meaningful future research about disability [22]. CDS researchers have a particular advantage over medical researchers when recruiting and encouraging individuals with disabilities to participate in research because CDS focuses on empowering the community and challenges structural and historic ableism. Together, interdisciplinary research teams that include CDS scholars can promote trustworthiness in relationships between disabled communities and medical researchers [36,37] and, ultimately, in findings from research.

### 4.2. Opposing Trainings

A key difference between CDS scholars and Healthcare and Medical Research scholars lies in their training. Individuals conducting research in Healthcare and Medical Research fields are instructed to diagnose and treat diagnoses. CDS researchers are trained to challenge systemic ableism and empower individuals with disabilities. In short, medical researchers take a medical approach to disability while CDS scholars take a human rights approach to disability.

Consider how the two medical members of our interdisciplinary team might approach research on individuals with Down syndrome diagnosed with early onset Alzheimer’s disease (EOAD). The medical professionals may be inclined to center their inquiry on an understanding of the pathological differences of this population, compared to those with EOAD who do not have comorbid Down syndrome, complications of differential diagnoses in the target population, and clinical trials, as has been the case in established medical research on the same populations [38]. Our two CDS researchers may voice concern that this approach is far too focused on diagnostics and take issue with the inherent ‘othering’ of the target population based on what they have predetermined to be ‘typical.’

Another difference between these fields can be found in the communication of science and new knowledge, as variations exist in ways that each research field articulates research, largely dependent upon who is accessing information in scientific publications [39]. When discussing findings and implications, Healthcare and Medical Research articles are often filled with complex language and jargon. In contrast, CDS research articles typically use accessible language intentionally understandable by the populations they serve. Differences in accessibility and language can create challenges when disseminating research findings to high-impact journals and directly to disabled communities. While arguments can be made for both styles of academic reporting, products of research studies from these two fields are inherently different, and as a result, reach different audiences.

Due to continued differences in points of view and education, it is challenging for the fields of CDS and Healthcare and Medical Research to work together. Cooperation is not always possible. Individuals conducting intersectional research should not have to compromise their core beliefs and epistemologies when designing studies and describing outcomes. However, incorporating multiple points of view and acknowledging differences in approaches to research on disability can propel negotiation towards what possible research projects can be produced by intersectional teams.

## 5. Possibilities

Bringing together contrasting fields or research can be challenging but also rewarding. Combining the fields of CDS and Healthcare and Medical Research creates the opportunity to generate new knowledge and deeper understanding of the complexity of human disability experiences. Bringing together various disciplines for rich, meaningful research does not require reinventing the wheel—frameworks for integrating interdisciplinary research teams already exist, including the Integrated Model of the Interdisciplinary Research Process (IRP; [40]). This approach emphasizes that the different parties trying to collaborate should clearly lay out their respective approaches, including supporting literature and theoretical perspectives. Then, the researchers can work together to recognize the value in other insights, identify and resolve conflicts, integrate ideas, and ensure that participants have a clear understanding of the research plan and goals. Considering the research team scenario and the challenges they may face in collaboration, we propose several possibilities to promote such collaborative communication and concordance.

### 5.1. Holistic, Person-Centered Research

From a research perspective, the person-centered approach (PCA) identifies the individual as an expert of their own experiences and characteristics, where the individual is self-directing, self-maintaining, and seeking to further understand and enhance their perception of self [41]. Integral to the original conceptualization of PCA is that, even though a particular experience might be the topic of interest for any given research project, each individual is a complex being whose experiences inform the target experience [42,43]. Essentially, as an expert on themselves, an individual can provide unparalleled insights into a particular experience and provide feedback on what research topics/methods might be most relevant and inform researchers on the intersectional nature of their life experiences.

In the context of uniting the medical model and a CDS approach to disability research, using a PCA fosters an important collaborative methodology. CDS place significant emphasis on equal accessibility for everyone [9] and inherently acknowledges the intersectional experiences of each person. Consequently, the individual is just as essential as the larger group in informing and facilitating the goal of CDS research, which is to promote wide-spread, universal accessibility. Medical researchers can also draw upon the intersectional experiences of individuals with and without disabilities to inform research on their health and wellness. By centering the individual in the research, medical researchers are afforded the opportunity to understand unique medical needs, promote individual agency, and shape medical practices and advances, rather than project medical advances onto the larger group.

### 5.2. Inclusive Participation and Co-Production

Developing and employing unique research approaches and practices can help bridge the gap between the two CDS-trained researchers and the two physicians in the scenario and guide a more effective and holistic research approach. Action research emphasizes the agency of the individual as an active participant in research processes to facilitate improvements to their daily lives and experiences [44]. In particular, Kemmis et al. (2013) [44] identify that a defining characteristic of Critical Participatory Action Research (CPAR) research is the following:

*Participants in social and educational life can do research for themselves. Others may also research social and educational life, but participants have special access to how social and educational life and work are conducted in local sites by virtue of being ‘insiders’ [p.5].*

Essentially, CPAR encourages the individual to define and lead research based on their own experiences, with academic researchers supporting them, following their cues, and recording processes and outcomes. Our research team might consider a qualitative study by Scott et al., (2014) [45], where researchers met with young adults with Down syndrome for both one-on-one and group interviews. One of the primary goals of their study was to understand participants’ perspectives on different facilitators of and barriers to their general well-being. Through these interviews, researchers realized the need to align research of medical advancements meant to increase the lifespan of those with Down syndrome with an investment in understanding and facilitating quality of life based on direct feedback from participants [45]. Individuals within populations of interest are ready and willing to engage in research and to work with researchers to generate meaningful, relevant ideas regarding social autonomy and rights as well as clinical needs and experiences, serving as a point of reference to inform collaborative research between our CDS and medical researcher team.

The research team might also consider engaging more formal community stakeholders as a mechanism to inform the direction of the research and identify needs. A particularly pertinent example of this type of positive community stakeholder can be found in the presence of networks of community health workers (CHWs) and the populations they serve. CHWs are members of the community whose primary roles are to facilitate the accessibility of medical education and information to surrounding communities and serve as a mediator between the community and larger systems [46]. While CHWs are often trained in collaboration with various medical systems, CHW roles are often intentionally and inherently interdisciplinary in their training and purpose [47].

It is already documented that CHWs report engaging in community advocacy by communicating community needs and asking for assistance to meet those needs directly (e.g., in-person conversation, letters, etc.) to elected officials, healthcare systems, and social services [48], even though this may be outside of their scope of duty. As such, many CHWs are familiar with the experiences and needs of the community they serve and how to communicate those experiences and needs to other individuals and organizations. Inclusion of CHWs in disability research may also offer unique benefits to the success of research.

CHWs are often embedded and respected members of the community that they serve [49] and often engage with underserved communities, many previously harmed by or have reason to be wary of the medical system or research [46]. Because of their experience as bridges connecting communities and individuals to other resources in the context of public health, incorporating CHWs has the potential to facilitate a more positive relationship between individuals, the researchers, and the wider community, boosting participation and feedback and ultimately adding important perspectives for interdisciplinary research teams [50]. CHW’s engagement in advocacy, commitment to disseminating information and resources to entire communities, and emphasis on social determinants of health [47] is well-aligned with the values of CDS researchers. CHWs, especially those serving in clinical roles, could be mobilized as collaborators in research to act as a liaison between disabled communities and researcher thus bridging gaps in CDS and medical model research.

### 5.3. Actionable Guidance for Collaboration in Practice

Communication will be key throughout collaborations and projects, but establishing expectations at the beginning is crucial to a successful and equitable experience for researchers and community members. Research teams might draw from collaborative frameworks such as the aforementioned IRP, or from current standards of collaborative research outlined by a bodies like the American Psychological Association [51]. Depending on the project, communication at the outset of the project should involve:

1. A mutual agreement on and understanding of the study goals and what the motivation of the research is.
2. What the desired outcome(s) of the research is.
3. Identifying differing points of view and ideas and working to resolve them through compromise.
4. Clearly stating the role each person will play (e.g., primary investigator, community liaison, data manager, etc.) and clearly outlining responsibilities.
5. Determining logistics of data collection: who will be recruited, how will they be recruited, what measures will be used, where will data be stored and managed, etc. Also, another thing to consider is who will primarily be in contact with participants: the researchers or the community experts?

These discussions should be kept in writing or recorded, and diseminated to all parties after each meeting. Record-keeping in this way can also serve to track how the project has evolved over time and hold all parties responsible for their contribitions. Teams should have regular check-ins over the course of the project, as research projects often evolve over time. Record-keeping in both in writing and auditory format is a good example of accessibility in the research process—using multiple formats and strategies to increase accessibility to all team members. Research teams should work to identify the needs of all members to ensure an experience that is accessible to all team members. This may include different ways of communication (written form, recording for revisiting later, real-time employment of an American Sign Language (ASL) translator, etc.) or different modes of attending research meetings or engaging in active data collection (transportation services for in-person meetings, options for joining via Zoom, etc.).

Individuals with disabilities, disabled communities, and community stakeholders should be considered experts and team members, not just consultants. This is particularly important in disability research collaboration, where participants and collaborators have historically been exploited and researchers have taken all credit. As such, the following should be established from the outset of a collaboration:

1. Regarding input from non-academic collaborators as equally valuable and actionable.
2. Formal credit for their contributions, including in project proposals, grant applications, and on publications and presentations.
3. Compensation as appropriate—for example, in the case of funded research, all collaborators should be compensated for their time and effort, not just the academic and/or medical researchers.
4. Ownership of the works—often times, the ‘ownership’ of research and data belongs to the funding body or the larger institution of the primary researchers. While this may be difficult to navigate, every effort should be made to ensure non-academic collaborators maintain access and rights to the data and research.

There are many other parts of the collaborative research process, and the afforementioned strategies are not exhaustive. However, communication is the bedrock of successful, accessible, and ethical collaborative research.

## 6. Conclusions

If the shared goal of any research team is to best serve the wants, needs, and interests of the population on which the research is focused, then an interdisciplinary research team can contribute enhanced depth to their fields by working together to create new knowledge. Using the hypothetical framework of a mixed research team as discussed above, different philosophical, methodological, and conceptual questions and ways of thinking can be addressed when bringing together contrasting research fields such as CDS and Healthcare and Medical Research. Future researchers who seek to join CDS and health fields should utilize creative research methods and intentionally include disabled partners and participants in every aspect of the research project. Engaging in participant- and community-centered research that supports the voices and experiences of disabled communities will increase rigor in research findings and trustworthiness in the partnership between researchers and disabled populations. Spending time in the gray/messy areas may be uncomfortable or difficult, but those are the precise areas where having multiple points of view and types of training will best serve the individuals and outcomes of research. Thinking critically and being transparent and descriptive throughout the research process enables opportunities to best serve disabled communities through research.

## Data Availability

The original contributions presented in this study are included in the article. Further inquiries can be directed at the corresponding author.

## Abbreviations

The following abbreviations are used in this manuscript:

CDS	Critical Disability Studies
EOAD	Early Onset Alzheimer’s Disease
PCA	Person-Centered Approach
CPAR	Critical Participatory Action Research
CHW	Community Health Workers
